# A benchmark of hemoglobin blocking during library preparation for mRNA-Sequencing of human blood samples

**DOI:** 10.1038/s41598-020-62637-0

**Published:** 2020-03-27

**Authors:** Florian Uellendahl-Werth, Markus Wolfien, Andre Franke, Olaf Wolkenhauer, David Ellinghaus

**Affiliations:** 1 Institute of Clinical Molecular Biology, Christian-Albrechts-University of Kiel, Kiel, Germany; 2 Department of Systems Biology & Bioinformatics, University of Rostock, Rostock, Germany; 3 Stellenbosch Institute of Advanced Study (STIAS), Wallenberg Research Centre at Stellenbosch University, 7602 Stellenbosch, South Africa

**Keywords:** Molecular medicine, Bioinformatics, Gene expression analysis

## Abstract

RNA-Sequencing (RNA-Seq) of peripheral blood can be a valuable source of information for investigating the status and mechanism of diseases. However, blood contains 50–80% unwanted hemoglobin (Hb) transcripts. Lexogen’s QuantSeq mRNA-Seq-Kit for Illumina RNA-Seq features a ‘Globin Block’ (GB) module that depletes Hb cDNAs during library preparation. Here, we aimed to assess GB’s effectiveness and checked for technical biases attributable to GB. Using whole blood total RNA samples of 91 healthy individuals, we sequenced 91 pairs of GB and non-blocked samples (noGB) on Illumina HiSeq2500 and 8 pairs of GB/noGB technical replicates on HiSeq4000. GB reduced the fraction of Hb transcripts from 43% (s.d. 14%) to 8.0% (s.d. 4.3%). From GB samples we detected 1,397 more expressed genes at approximately 11 million reads per RNA-isolate. Enrichment and differential expression analyses did not reveal significant differences for GB and noGB samples with respect to molecular function. In contrast to results from studies that have examined the performance of GB during RNA isolation, we were able to assign GB to corresponding noGB samples (from multiple sequencing runs on HiSeq2500) with at least 89.8% accuracy from the complete correlation matrix of all GB/GB, noGB/noGB and GB/noGB pairs. However, the use of different sequencers (HiSeq2500 vs HiSeq4000) impaired assignment of technical replicates, whereas assignment of GB to corresponding noGB samples worked perfectly when sequencing on one lane on HiSeq4000. Lexogen’s GB RNA-Seq module is a valuable addition during mRNA-Seq library preparation which works even with low amounts of input total RNA (50 ng per sample). GB facilitated the detection of low abundant transcripts and yielded more non-hemoglobin reads, while preserving biological information. We observed that differences in sequencing run and platform have a far greater effect on technical variation than the use of GB.

## Introduction

RNA-Sequencing (RNA-Seq) has become an affordable and accurate molecular method to quantify transcripts and to detect yet unknown RNA species^1,2^. So far, RNA-Seq applications have been shown to predict relevant clinical outcomes^3^ and are increasingly used in diagnostics to personalize treatments^4^, but also led to new insights into transcriptional biology^5–8^. Peripheral blood is widely used in clinical research and diagnostics since it is an easily accessible connective tissue, which is closely related to the status of the body^9–13^. However, RNA-Seq of whole blood samples is impeded by the abundance of Hb transcripts, that finally take up 50–80% of the sequenced reads^14,15^ and researchers are usually interested in the remaining 20–50% of non-Hb transcripts, as these include potential biomarkers. The high abundance of Hb transcripts, in particular HBB-201 (ENST00000335295) and HBA2-201 (ENST00000251595), reduces the reliability to quantify low-expression transcripts^14^ and, therefore, requires deeper sequencing with higher read amounts, which quickly becomes costly.

To overcome this limitation, depletion kits have become commercially available to selectively remove Hb transcripts before sequencing. Technically, there are two different approaches: The standard approach removes globin mRNAs from the RNA isolate by hybridizing with nucleotides ligated to magnetic beads^16,17^. This hybridization approach has already been shown to improve the ability to detect true biological variation but leads to significant reduction in RNA yields and manipulates the sample itself during RNA extraction, i.e. before library preparation and sequencing^14,18–21^. Recently, a new approach called GlobinLock^15^,which preserves RNA quality of the original sample, has been introduced that selectively blocks the second strand synthesis of the globin-cDNA and was further developed commercially by the company Lexogen with the promise to reduce globin mapping reads by up to 91%^22,23^. This second approach has been shown to work well with porcine blood samples^24^, but so far no thorough evaluation has been conducted for human blood samples. Here, we investigated whether Lexogen’s mRNA-Seq Kit featuring the new GB module is a worthy alternative to established globin reduction kits, which manipulate the original sample during RNA extraction. We evaluated data quality from 91 RNA-sequenced human peripheral blood samples on Illumina HiSeq2500, all prepared once with GB and once without GB (referring to as *noGB*), also with a focus on biological meaningfulness and integrity. By means of technical validation (Illumina HiSeq4000), we checked for reproducibility of our results.

## Results

### GB yielded 60% more (non-Hb) reads and improved unique alignment accuracy by 17%

**Figure 1:**
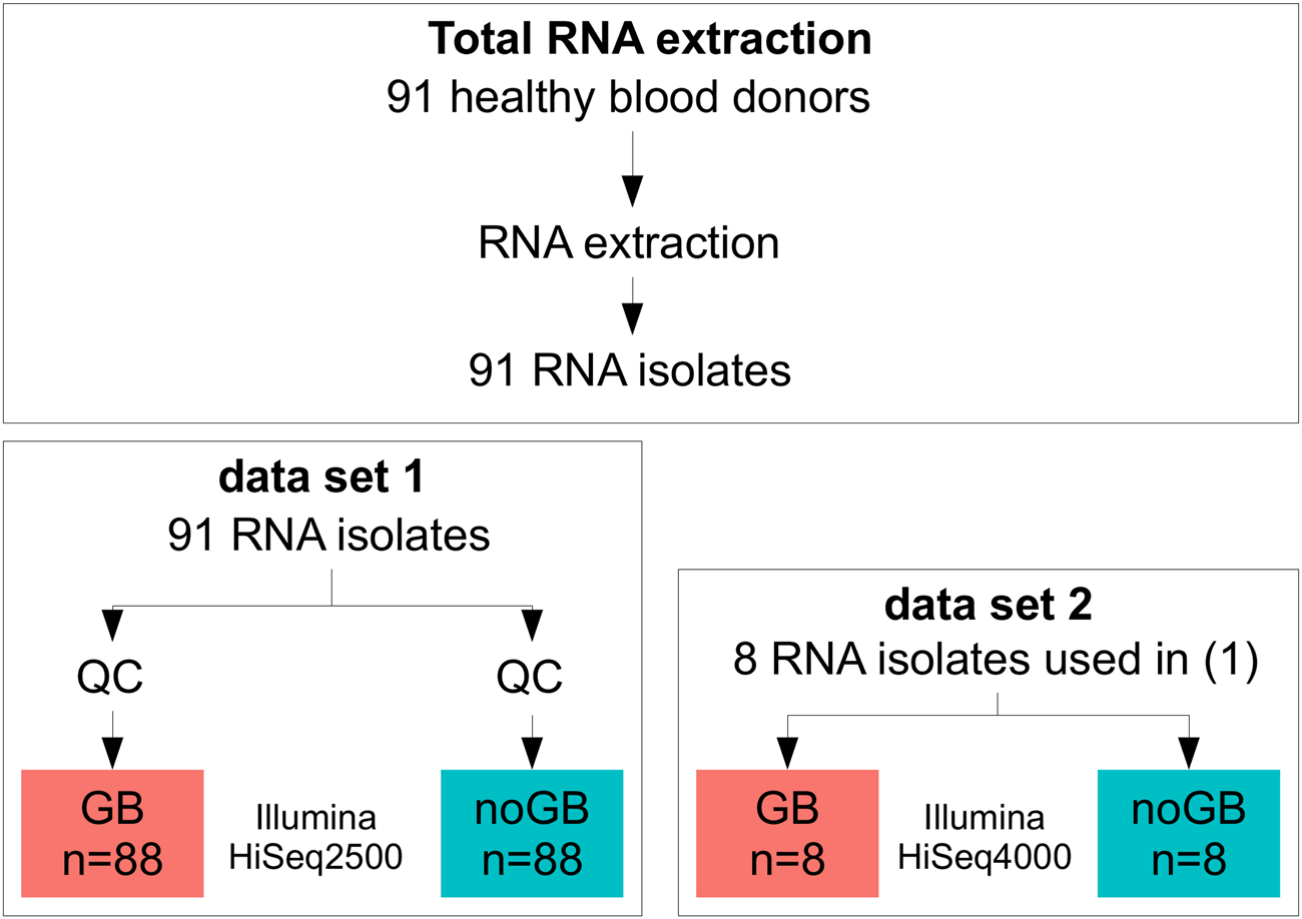
Study design to test the performance of Lexogen’s commercially available globin-cDNA blocking method that was applied during NGS RNA library preparation (thus preserving RNA quality of the original sample). Total RNA was isolated from whole blood samples of 91 healthy donors (see Methods). For data set 1, two libraries were prepared from all 91 RNA isolates, i.e. one with Lexogen’s Globin Block (GB) and one without (noGB), followed by sequencing on the HiSeq2500 instrument. After quality control (QC), 88 pairs of GB/noGB samples were available for benchmark purposes. For data set 2, 8 GB and noGB samples from Data Set 1 were re-sequenced on a HiSeq4000 to assess technical reproducibility for GB samples.

**Figure 2:**
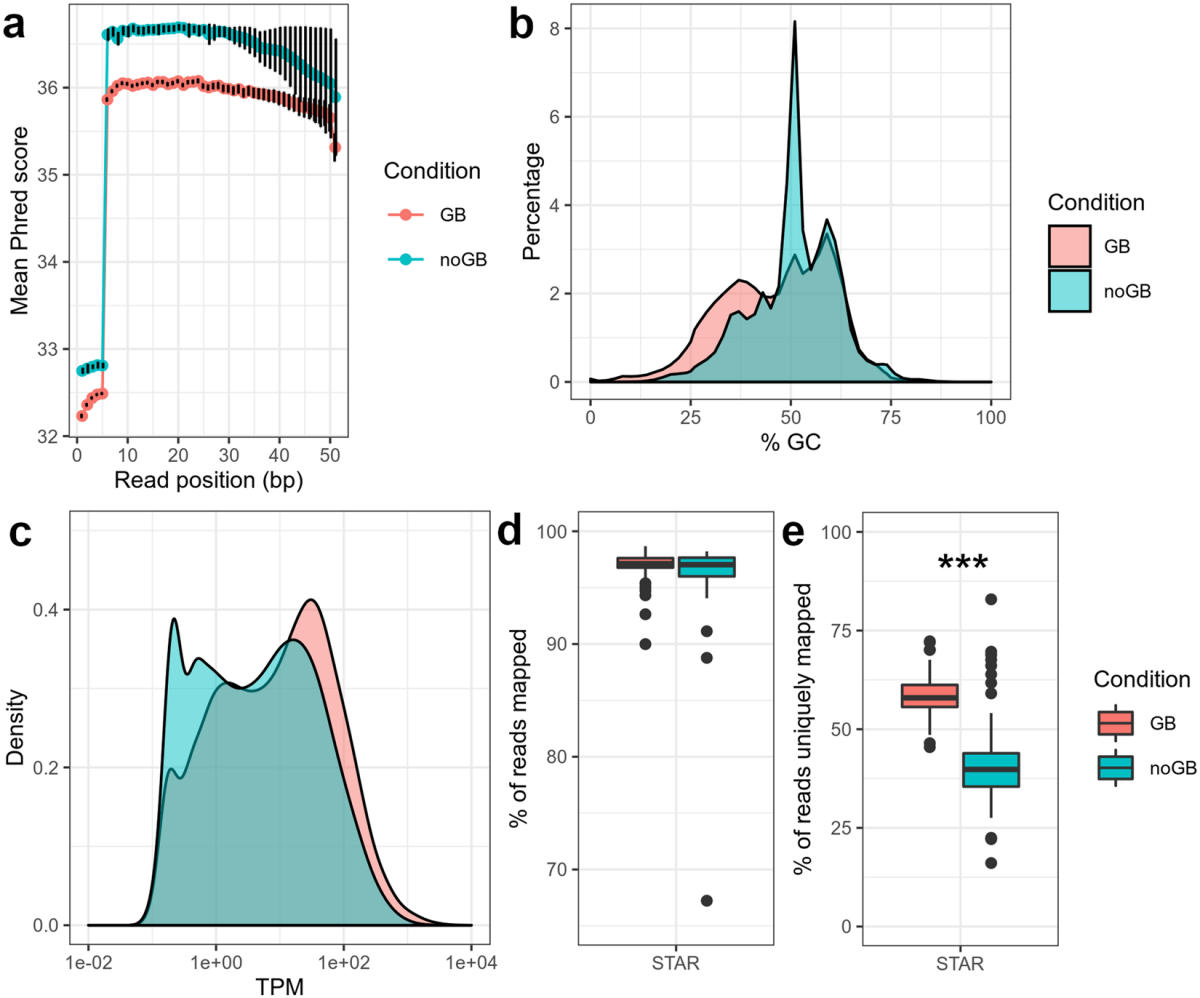
Globin block (GB) samples showed superior effects over noGB on various RNA-Seq quality and quantity measures. (**a**) Although read quality as reported by the sequencer is slightly reduced in GB compared to noGB, the effect is negligible. For example, a reduction of approximately 0.5 in Phred score Q equals a base-calling error probability increase from 2.45e-4 to 2.75 × 10^−4^. (**b**) The spike of reads with 51% GC-content vanished in GB samples since 3’-biased Hb transcripts HBB-201 are known to have a GC-content of 51%. (**c**) The expression density distribution is shifted towards more (non-Hb transcript) reads for GB samples, yielding 1,397 more unique genes for subsequent differential expression analysis. TPM: transcripts per million. (**d**,**e**) While the overall alignment rate is consistent between GB and noGB (**d**), the unique mapping rate increased by 17.1% for GB (58.3%) as compared to noGB (41.2%) (***p < 0.001, two-sided t-test) (**e**).

First, we investigated whether GB had a significant effect on common RNA-Seq quality and quantity measurements. For this, we used 88 samples after quality control (QC; see Methods), of which one library was prepared with Globin Block and the second one without Globin Block, followed by RNA-Seq with eight lanes on the HiSeq2500 (data set 1; see Methods; Fig. 1). The total number of reads in data set 1 was significantly lower with GB as compared to noGB (GB: 11.3 million, standard deviation (s.d) 2.8 million, noGB: 13.8 million, s.d. 2.1 million, two-sided t-test *p* < 0.001). This is not surprising, because in the GB sequencing library preparation step, second cDNA strand syntheses were aborted due to the GB nucleotides. To some extent, the number of reads may be increased by additional PCR cycles or higher input of RNA, but users should pay attention to possible PCR bias often occurring during the last few cycles^25^. The quality of reads was slightly better in noGB samples compared to GB samples but with negligible effect (average Phred score Q (e.g. Q = 30 equals 0.1% error probability), noGB: Q = 36.1, GB: Q = 35.6, two-sided t-test, *p* < 2.2 × 10^−16^, Fig. 2a). We investigated the distribution of GC content for GB and noGB samples as an indicator of the success of globin depletion. The averaged GC distribution for GB samples showed a peak at 51% (Fig. 2b), which could be caused by the over-abundant HBB-201 transcript that shows a GC content of 51% on average including the 3′-UTR (HBB-201, www.ensembl.org, release 97). Interestingly, GB samples had a significantly higher number of duplicated reads as compared to noGB samples (GB: 74%, s.d. 6.5%, noGB: 71%, s.d. 9.3%, two-sided t-test, *p* = 0.016). This was unexpected since high duplication levels for sequences predominantly originate from highly expressed genes, such as globin transcripts. Next we compared the distribution of TPM values between GB and noGB samples for 37,423 genes, coding and non-coding, which were counted at least once in any sample. We counted 1,397 more unique genes with average transcripts per million (TPM) > 1 for GB samples (16,148 genes) compared to noGB samples (14,751 genes), with ∼11 million reads per RNA-isolate (Fig. 2c, Supplementary Fig. S1). The fraction of globin transcripts was reduced from initially 43% (s.d. 14%) to 8.0% (s.d. 4.3%) by using GB (Supplementary Fig. S2), which means that the number of non-globin reads was increased by 60% per mapped read. While the overall alignment accuracy was similar between GB and noGB (GB: 97.0%, s.d. 1.1%, noGB: 96.3%, s.d. 3.4%, two-sided t-test, *p* = 0.07, Fig. 2d), the unique mapping rate was significantly improved by GB (GB: 58.3%, s.d. 5.1%, noGB: 41.2%, s.d. 10.6%, two-sided t-test, *p* < 2.2 × 10^−16^, Fig. 2e) probably due to the removal of highly similar Hb transcripts.

### GB and noGB samples show similar TPM values for non-Hb transcripts

**Figure 3:**
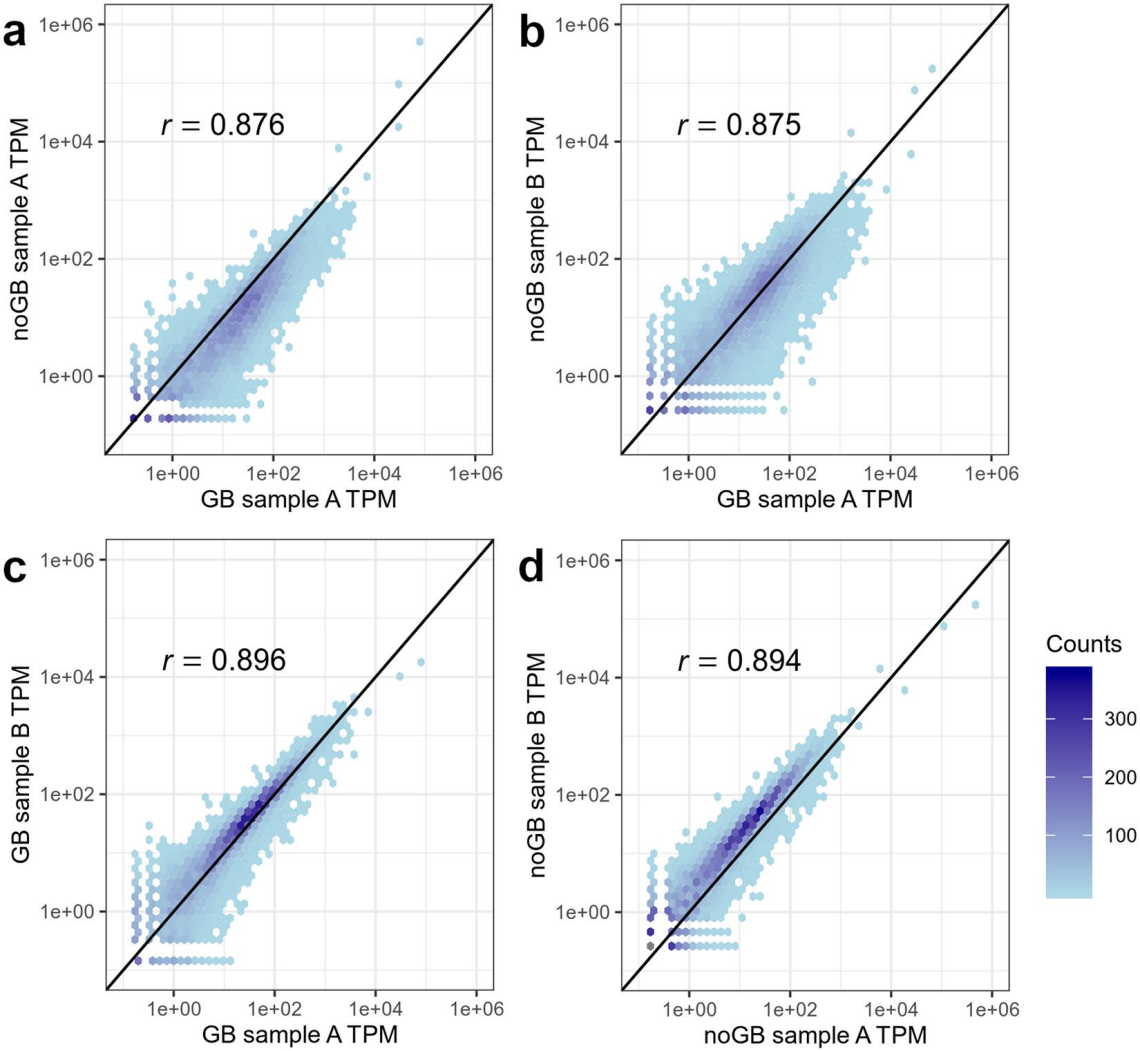
Potential batch effect introduced by “sequencing run” (i.e. using different flowcells on HiSeq2500 for GB and noGB samples) led to lower correlation between GB and noGB samples from the same RNA-isolate as compared to pairs of GB/GB and noGB/noGB from different RNA-isolates. Spearman correlation between GB/noGB gene expression from **(a)** the same RNA-isolate (i.e. same individual) and (**b**) different isolates (i.e. different individuals) was almost equal. Interestingly, pairs of GB/GB **(c)** and noGB/noGB **(d)** derived from different individuals showed higher correlation than biologically identical samples **(a)**. TPM: transcripts per million.

To find out how similar TPM values of non-Hb transcripts are with and without use of GB, we calculated Spearman correlation on the transcriptome-wide scale for all possible pairs of GB/noGB samples from same individuals (i.e. same RNA-isolate) as well all possible pairs of GB/GB, noGB/noGB and GB/noGB samples from different individuals (i.e. different RNA-isolates) in data set 1 (Supplementary Fig. S3). Averaged over GB and noGB samples from all 88 individuals, we observed a trend that GB samples from different individuals are higher correlated to each other than to their respective paired noGB sample from the same RNA-isolate (GB samples: 0.916, paired samples: 0.907, *p* = 0.007, two-sided t-test). For demonstration purposes, we randomly picked out GB/noGB samples of two individuals (Fig. 3). Notably, Spearman correlation for pairing GB and noGB samples from different individuals was nearly the same (example r = 0.875, average r = 0.874, s.d. 0.079; Fig. 3b) compared to the correlation between GB and noGB sample from the same individual (example r = 0.876, average r = 0.907, s.d. 0.027; Fig. 3a). Note that the correlation of samples from different individuals would be expected to be higher, if outlier samples were removed, which are shown as white columns in Supplementary Figure S3. Interestingly, correlation values between GB samples from different individuals (example r = 0.896, average r = 0.916, s.d. 0.090; Fig. 3c) and between noGB samples from different individuals (example r = 0.894, average r = 0.927, s.d. 0.086; Fig. 3d) were notably higher. We assume that the slightly lower correlation between GB and corresponding noGB data from same individuals is due to “sequencing run” batch effects (i.e. GB and noGB samples from same RNA isolates were sequenced on different runs (each with four lanes) on HiSeq2500, see Methods); this impressively shows the downside of combining RNA-Seq data from different experimental settings or sequencing runs.

### GB and noGB samples from identical RNA-isolates matched perfectly for 79 out of 88 individuals

**Figure 4:**
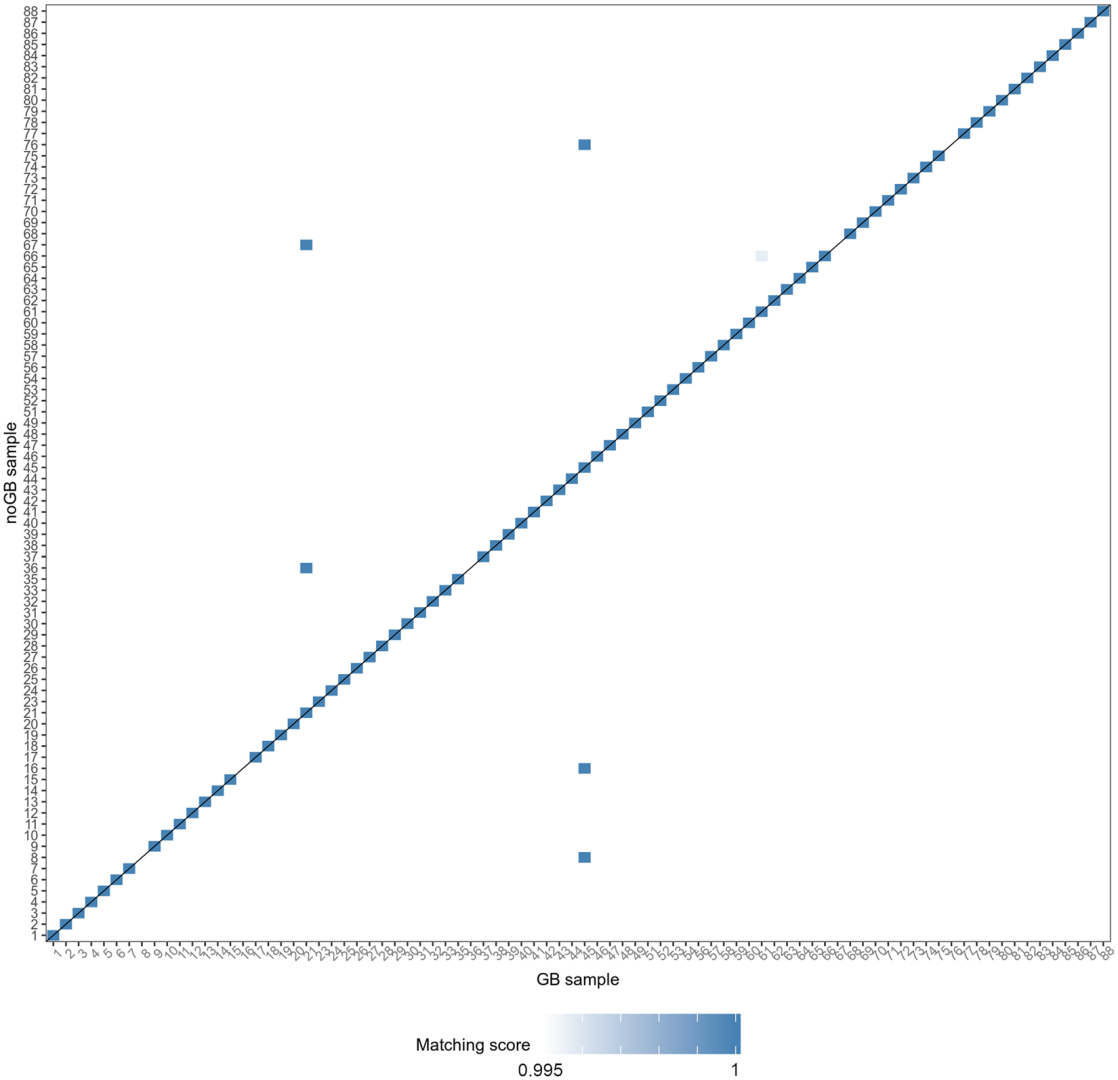
Scaled Spearman correlation analysis (see Methods) showed that GB does not impair assignment of biologically identical samples in 89.8% of cases. For 79 out of 88 individuals, a blue square on the diagonal represents a perfect assignment of GB and noGB samples from same RNA-isolate. For 5 out of 88 individuals (i.e. data points off from the diagonal), true GB/noGB pairs from the same RNA-isolate were not identified correctly. Note that we excluded 4 pairs of GB/noGB prior to final scaled Spearman correlation analysis, since these “outlier” samples caused multiple mismatches during a first round of Spearman correlation analysis (see Methods and Supplementary Fig. S4).

To examine whether GB sequencing libraries still contain most of the same transcript information as compared to noGB, we matched corresponding data sets from GB and noGB by means of scaled Spearman’s correlation analysis (Fig. 4, Supplementary Figures S4 and S5), i.e. scaling the raw correlation coefficient by the mean of the column and setting the highest value per row to 1 (see Eq. 1 in Methods section). Inspecting the raw Spearman coefficients (Supplementary Fig. S3), four samples were determined as outliers as they correlate far less with any other sample than most of the other samples. These four GB samples caused multiple wrong matches to noGB samples (Supplementary Fig. S4). After excluding these four pairs from initial analysis, we repeated the calculation of the Spearman’s correlation coefficient for the remaining 84 GB samples. Out of the 84 pairs, 79 matched correctly. When we removed the two samples (#21 and #45) causing mismatches in Fig. 3, only one assignment is false (Supplementary Fig. S5). In total, for 79 out of 88 individuals, GB and noGB samples from same RNA-isolates (i.e. biologically identical samples) matched perfectly (89.8%).

### Sequencing machine batch (HiSeq2500 vs. HiSeq4000) effect impaired assignment of technical GB replicates

**Figure 5:**
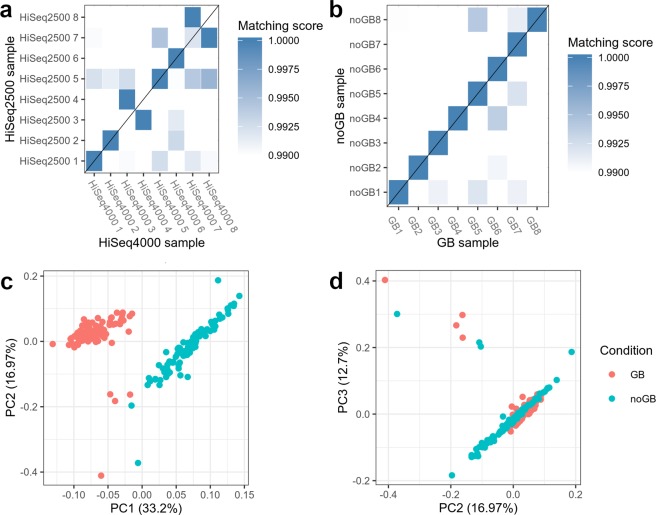
Sequencing machine batch effects [HiSeq2500 (data set 1) vs. HiSeq4000 (data set 2)] impaired assignment of GB technical replicates in comparison to perfect matching of pairs of GB/noGB samples from same RNA isolate [HiSeq4000 (data set 2)]. (**a**) A correct assignment of GB/GB samples (as well as noGB/noGB samples, not shown) from the same RNA-isolates was successful only for 4 out of 8 pairs of GB technical replicates, i.e. pairs #1, #2, 5, and #6. (**b**) Assignment was perfect for GB/noGB samples from same RNA isolate sequenced on one lane of a HiSeq4000. (**c**). Results from PCA for pairs of GB/noGB samples [HiSeq2500 (data set 1)] showed significant differences in the first component. (**d**) The second and third component were more balanced between GB and noGB samples (data set 1; both HiSeq2500).

We further compared the correlation pattern of TPM values with technical replicates of GB samples from same RNA-isolates (data set 2; n = 8 samples, Fig. 1; see Methods) to assess the technical reproducibility of GB results. These samples could be affected by potential sequencing machine batch effects (HiSeq2500 vs. HiSeq4000). When trying to match GB samples of data set 1 and 2 to each other, surprisingly, we found that the sequencing machine effect impaired the correct assignment of technical GB replicates, with only 4 out of 8 correct matches after scaled Spearman correlation analysis between technical replicates (Fig. 5a). Raw Spearman correlations between HiSeq4000 and HiSeq2500 samples were still high (average r = 0.908, s.d. 0.013, Supplementary Fig. S6). Principal component analysis (PCA; see Methods) showed a clear difference in the first principal component between the HiSeq2500- and HiSeq4000-sequenced GB samples (Supplementary Fig. S7). Since all 8 pairs of GB/noGB samples from same RNA-isolates were sequenced on one lane of the HiSeq4000 (see Methods), this experimental setting was expected to exclude any lane or platform bias between GB/noGB samples of data set 2. Indeed, all 8 pairs of GB/noGB samples matched correctly (Fig. 5b), and PCA (Supplementary Fig. S8) and raw Spearman correlation coefficient heatmap results across all possible GB/noGB pairs (Supplementary Fig. S9) revealed highest correlation for GB/noGB samples of same RNA isolates. These results clearly show the experimental bias from different sequencing experiments. Therefore, we conclude that some bias introduced by GB in comparison to noGB, if any, is of a limited extent compared to the bias that is introduced by the use of different types of sequencing machines or running on different flowcells.

### No significant differences between GB and noGB data sets with respect to molecular function

Since we observed the trend that GB and noGB samples, respectively, from different individuals (Fig. 3c,d) were higher correlated to each other than to their respective paired noGB sample from the same RNA-isolate (Fig. 3a), we aimed to determine the genes causing the difference between GB and noGB in data set 1. Since the first three principle components (with Hb transcripts removed beforehand; see Methods) already contained 62.9% of the variation with gene-based TPM counts (PCA plots, see Fig. 5c,d), we selected each the top 100 genes with highest absolute gene loadings (Supplementary Table S1) from first three principle components (PCs) from the PCA in order to investigate their molecular function by means of gene set enrichment analysis using EnrichR^26,27^ and data bases from Human Gene Atlas and Gene Ontology (see Methods). Instead of intending to generate clear biological results by means of enrichment analysis, we performed enrichment analysis to investigate for potential false positive biologically functional results when contrasting expression data generated from GB and noGB experiments. In concordance with the observation that GB and noGB samples are clearly separated for the first PC, enrichment analysis revealed 34 (0; 9) significantly enriched (adjusted *p* < 0.05) Human Gene Atlas or gene ontology (GO) terms for the first (second; third) principal component, among others, related to terms such as B-lymphoblasts, telomers, RNA binding and processing (Supplementary Table S2). However, these terms are likely to be false positives, because our “negative control” EnrichR analysis using the list of all 13,493 genes available from PCA (average and median TPM > 1) was also significantly enriched (adjusted *p* < 0.05) for 345 out of 6784 terms in total, of which 14 were shared with principal component 1 (see tabs “all_PCA_genes”, Supplementary Table S2). The probability of having 14 or more shared terms out of 34 is very unlikely (*p* = 3.29 × 10^−10^; hypergeometric test) showing that top 100 genes (in comparison to all input genes) from PCA are unlikely to be enriched with genes biased towards particular biological functions.

As a next step, we prioritized genes by means of differential expression analysis with DESeq2^28^. We selected genes which were strongly downregulated in GB compared to noGB samples (log2fold-change < −3, p-adjusted <0.05, Supplementary Table S3) and excluded all hemoglobin genes (*HBA1*, *HBA2*, *HBB*, *HBD*, *HBE1*, *HBG1*, *HBG2*, *HBM*, *HBQ1*, *HBZ*). Because it is counterintuitive that genes except hemoglobins appear downregulated in GB but not in noGB samples, we checked if these genes were significantly enriched with EnrichR; we didn’t observe any significant result (see tabs “DEG”, Supplementary Table S2).

### Blocking of globin transcripts is unlikely to affect non-globin transcripts

Finally, we assessed whether GB might have an effect on non-globin transcripts due to sequence similarities between globin and non-globin transcripts. For each of the first three principal components from PCA on data set 1, we checked for mRNA sequence similarities of the top 100 variance-conveying genes’ cDNAs to hemoglobin cDNAs using blastn^29^. None of the cDNAs from ensembl release 98 aligned significantly with hemoglobin cDNAs (*HBA1*, *HBA2*, *HBB*) (Supplementary Table S4). Further, cDNAs of genes downregulated in GB as identified by DESeq2 (Supplementary Table S4), of which 212 out of 296 were non-protein-coding genes, did not show any significant sequence alignment with blastn. We conclude that sequence similarity with hemoglobin genes was not responsible for the occurrence of the PCA loading’s top 100 genes or differentially expressed genes.

## Discussion

In summary, Lexogen’s globin blocking (GB) module improved the quality (increased unique alignment accuracy of 17%) and quantity (60% more non-globin sequence reads) of our peripheral whole blood RNA-Seq data sets. Hemoglobin transcripts of genes *HBB* and *HBA2* were reliably depleted. By reducing only Hb transcripts in GB samples, we were able to detect 1,397 more genes (compared to noGB) at low abundance level (TPM > 1). Enrichment and differential expression analyses showed no evidence of a change in the biological information of GB samples. Differential expression analysis only revealed differentially weakly expressed pseudogenes, so that we assume that technical and biological noise is the reason for these results (possibly due to batch effect introduced by using different flowcells on HiSeq2500 for GB and noGB samples of data set 1). These results are in concordance with previous globin depletion benchmark studies on standard approaches that remove globin mRNAs from the RNA isolate itself (and not during library preparation). For example, Mastrokolias *et al*.^20^ reported pseudogenes predominantly downregulated in GB samples compared to noGB samples. Further, PCA loading analysis did not reveal features that could be attributed to a GB versus noGB library kit bias, and there is no evidence of sequence similarity between globin and non-globin transcripts among top hits of PCA-leading or differentially expressed genes’ cDNA. This result is in line with Lim *et al*.^24^ who, at the same time as our study, conducted a study using Lexogen’s Globin Block method for pig whole blood samples, in which they performed sequence similarity analysis on genes that were counted in noGB but not in GB samples, and found none. Interestingly, our result that GB in human samples significantly improves the unique mapping rate for sequence reads by 17.1% [GB (58.3%) as compared to noGB (41.2%)] of all aligned reads (probably due to the removal of highly similar Hb transcripts from HBB and HBA2, see also Supplementary Fig. S2; thus, more reads should be available from GB experiments for analysis at equal sequencing depth) is inconsistent with the results for porcine samples from Lim *et al*.^24^, who found the opposite [i.e. increase of unique mapping rate by 10.6% for noGB (78.3%) as compared to GB (67.7%)] and showed that GB substantially reduced only the amount of HBB reads relative to non-globin reads. We therefore hypothesize that, for human blood samples, GB works well for *HBB* as well as *HBA2*.

As in previous RNA-Seq benchmark papers^14,20,24^ we calculated Spearman correlation coefficients between all pairs of GB and noGB samples from the same RNA-isolate in order to prove that GB and nonGB samples match by high correlation coefficients. This was used as evidence to support that globin depletion causes only minor technical bias. However, Mastrokolias *et al*.^20^ found out that, when using Spearman correlation coefficients, GB samples from different isolates also correlated strongly with each other and did not match corresponding GB and noGB samples from same isolate from a full matrix of raw correlation coefficients from all GB and noGB samples. We addressed this problem by introducing scaled Spearman correlation analysis (see Methods). This allowed us to extend the matching analysis to all GB and no GB samples and to efficiently discriminate between GB and noGB samples from different isolates. For pair-wise matching analysis, we suggest evaluating the complete correlation matrix of all samples by means of scaled Spearman correlation analysis in future benchmark studies. Our matching procedure (see Methods) also worked well in the situation of a potential technical bias introduced by sequencing GB and noGB samples on different lanes but within same run on HiSeq2500 (data set 1). Since sequencing of 8 GB samples (technical replicates) on one single lane on HiSeq4000 resulted in lower correlation between all pairs of GB samples, all 8 GB samples could be perfectly matched to corresponding noGB samples from same RNA-isolate. Therefore, we concluded that differences in sequencing run and platform have a far greater effect on technical variation than the use of GB.

The main advantage of Lexogen’s GB solution is that it is easily employed during library preparation and does not reduce the RNA sample volume itself, as compared to common depletion kits that are employed during the RNA extraction preparation step. Further it works with low amounts of input total RNA (50 ng per sample) as compared to common depletion kits that consume 1.75–3 µg of human whole blood total RNA per sample^14^. Thus, we advocate the use of the GB module in combination with QuantSeq for RNA-Seq experiments of whole blood samples. At time of publication, we became aware of a competitive globin mRNA blocking product called QIASeq FastSelect, launched by QIAGEN in November 2019, that allows globin mRNA blocking during library preparation. Therefore, future benchmarks of globin blocking should include blocking modules from different vendors to compare their effectiveness as well as advantages and disadvantages of different blocking methods that can be used during library preparation.

## Materials and Methods

### Study samples

All participants provided written informed consent and the study (including all experimental protocols) was approved by the ethics boards of the University Hospital Schleswig Holstein (UKSH), in agreement with the Declaration of Helsinki principles. All experiments were performed in accordance with relevant guidelines and regulations.

### cDNA library construction and RNA-Sequencing

Total RNA, including small RNAs, was isolated from 91 whole blood samples of healthy blood donors using the PAXgene Blood miRNA Kit (Cat No./ID: 763134) from Qiagen. All RNA isolates had an RNA integrity number (RIN) value greater than 6. Two Lexogen QuantSeq3’ mRNA-Seq libraries were generated from each isolate, one applying the optional Lexogen GB module (Homo sapiens, Cat. No. 070.96) and one without any Hb blocking (i.e. noGB). The module solution of globin blockers simply replaces the standard RNA removal solution of the QuantSeq Kit in the GB experiment (https://www.lexogen.com/new-globin-block-modules-for-quantseq). In brief, after reverse transcription with poly-(T)-primers, the GB solution is added and specific GB oligos bind selectively to the globin mRNA cDNA-transcripts. The second strand synthesis is initiated by random priming. Because the globin blocking oligo is bound close to the poly-(T)-section of the first strand, second strand synthesis stops for globin transcripts and does not reach the 5′ sequencing tag of the first strand, thus yielding non-amplifiable globin cDNAs. During subsequent PCR cycles, only non-globin tagged double-stranded cDNA library fragments are amplified to add full-length adapters and indices for Illumina sequencing.

Lexogen QuantSeq3′ mRNA-Seq libraries for Illumina sequencing were generated using 50 ng of RNA and 15 PCR cycles. Single reads of a length of 50 base pairs (bp) were sequenced on an Illumina HiSeq2500. 91 pairs of GB/noGB samples were sequenced in two separate runs (each using four lanes) on the HiSeq2500 (data set 1). To check for reproducibility of GB samples, eight RNA-isolates were again sequenced including the GB Module but on an Illumina HiSeq4000 sequencer (data set 2). These samples were technical replicates, except for the fact that they were sequenced on two different sequencing machines (HiSeq2500 vs. HiSeq4000) in order to check for potential batch effects introduced by the sequencer and by splitting samples on different lanes. An overview of the study design is shown in Fig. 1.

### RNA-Sequencing data analysis

The raw, demultiplexed data was processed using usegalaxy.eu^30^, a European instance of the Galaxy data analysis framework, which facilitates bioinformatics workflows^30^. Our analysis workflow is publicly available at https://usegalaxy.eu/u/f-uellendahl-werth/w/starglobin-block. Quality control was conducted with FastQC (galaxy tool version 0.71) and MultiQC (galaxy tool version 1.6). Sequence reads were aligned with STAR^31^ (galaxy tool version 2.5.2b-2, defaults) against the built-in GRCh38.87 genome. Gene expression was calculated and quantified on the gene level as raw counts and transcripts per million (TPM) with HTSeq-count^32^ (galaxy tool version 0.9.1). During quality control, 3 GB/noGB pairs out of the 91 individuals were sorted out due to quality issues (too few reads, e.g. 0.2 Mio reads compared to ~11 Mio reads and outliers, identifiers in GEO: GSM3926757, GSM3926758, GSM3926775, GSM3926848, GSM3926849, GSM3926866).

### Scaled spearman correlation analysis

Finally, a total of 88 pairs of RNA-Seq samples (GB *vs*. noGB) were available for a direct comparison of GB and noGB samples. To assign each GB sample to the corresponding noGB sample from the same RNA-isolate we calculated a scaled Spearman’s (less sensitive than Pearson correlation to strong outliers) rank correlation score for pairs of GB and noGB samples (Eq. 1). The purpose of this score is to indicate, for a given sample, which other sample is the most similar one from another study group, like in our case, GB samples and noGB samples. Spearman correlation values of raw gene counts with an average TPM count >0.5 in both GB and noGB were computed in a matrix-like fashion for all noGB samples *vs*. all GB samples (*r*_S*i,j*_). For matching GB and noGB samples from identical RNA-isolates it was necessary to account for that some GB samples generally correlate stronger with noGB samples than other GB samples. Thus, each value was divided by the mean correlation coefficient of the respective column (${\bar{r}}_{j}$) (same GB sample *vs*. noGB samples) and then divided by the resulting maximum value of each row (*max*(…)). As a consequence, exactly one field per row (noGB sample) equaled one, and the other ones had values lower than one. Thus, if a GB sample and a noGB sample from the same RNA isolate had a scaled correlation score equaling one, they were correctly assigned.

1 $${r}_{modi,j}=\frac{{r}_{Si,j}}{{\bar{{\rm{r}}}}_{j}max{\left(\frac{{r}_{Si,j}}{{\bar{{\rm{r}}}}_{j}}\right)}_{i}}$$

### Analyes with respect to molecular function

Principal component analysis (PCA) was performed for (i) 88 noGB vs. the GB samples, (ii) 8 GB pairs of “technical” replicates (Illumina HiSeq2500 vs. Illumina HiSeq4000) and (iii) 8 noGB vs. GB samples on the HiSeq4000 on the same lane. The PCA was conducted on scaled TPM data. Genes for PCA analysis had to have a mean and median expression value (TPM) of at least 1.0 over all respective samples to be included. Genes were sorted by significance based on absolute values from transcript loadings of the first three components. DESeq2^28^, which estimates dispersion and performs differential expression analysis based on a Wald-test, was run in default settings on our count data. To investigate the most influential transcripts from PCA and differentially expressed genes with respect to molecular function we used EnrichR^26,27^, restricted to four databases Human Gene Atlas, GO Biological Process, GO Molecular Function and GO cellular component. EnrichR queries databases such as GO or Human Gene Atlas and performs overrepresentation analysis (ORA) on a set of input genes. To identify shared sequence features, sequence similarity of the top 100 influential genes’ cDNAs in comparison to the cDNAs of all transcripts of HBA1, HBA2 and HBB available on Ensembl Genes 98 were examined via blastn version 2.2.30 (word size = 6, E-value cutoff E < 1)^29^. Blastn is efficient at detecting similar base sequences in big nucleotide databases. The differentially expressed genes identified by DESeq2 were tested for sequence similarities in the same way.

## Supplementary information

Supplementary Figures.

Supplementary Table S1.

Supplementary Table S2.

Supplementary Table S3.

Supplementary Table S4.

## Data Availability

The datasets generated during and/or analysed during the current study are available in the Gene Expression Omnibus repository, https://www.ncbi.nlm.nih.gov/geo/query/acc.cgi?acc=GSE133758.
