# Supplementary material for: A benchmark of hemoglobin blocking during library preparation for mRNA-Sequencing of human blood samples
Source: Sci Rep. 2020 Mar 27;10:5630. doi: 10.1038/s41598-020-62637-0 (PMC7101437; doi:10.1038/s41598-020-62637-0)
Supplement: Supplementary file 1 — Supplementary Figures. [file 41598_2020_62637_MOESM1_ESM.pdf]

Supplementary Information for

A benchmark of hemoglobin blocking during library preparation for mRNA-Sequencing of human blood samples

Author list: Florian Uellendahl-Werth[1], Markus Wolfien[2], Andre Franke[1], Olaf Wolkenhauer[2,3], David Ellinghaus[1*]

[1] Institute of Clinical Molecular Biology, Christian-Albrechts-University of Kiel, Kiel, Germany
[2] Department of Systems Biology & Bioinformatics, University of Rostock, Rostock, Germany
[3] Stellenbosch Institute of Advanced Study (STIAS), Wallenberg Research Centre at Stellenbosch University, 7602 Stellenbosch, South Africa

* Corresponding author
E-Mail: d.ellinghaus@ikmb.uni-kiel.de

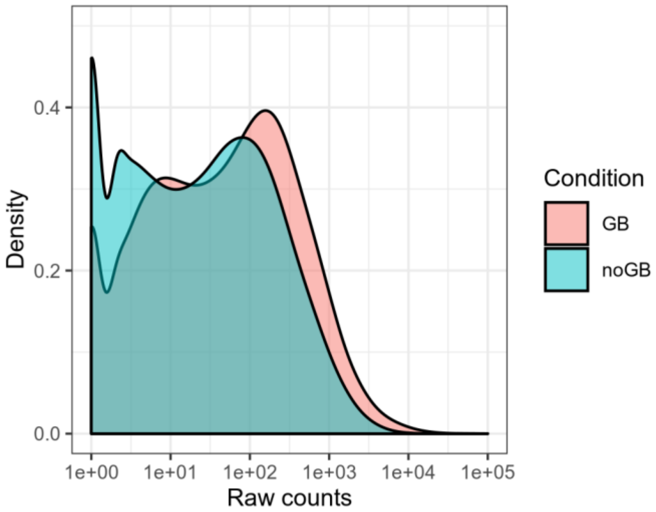

Supplementary Figure S1. The corresponding raw counts to the TPM values show the same pattern of more genes with higher count values. Note that, on average, noGB had a higher number of reads, so this diagram is unfavorable for GB.

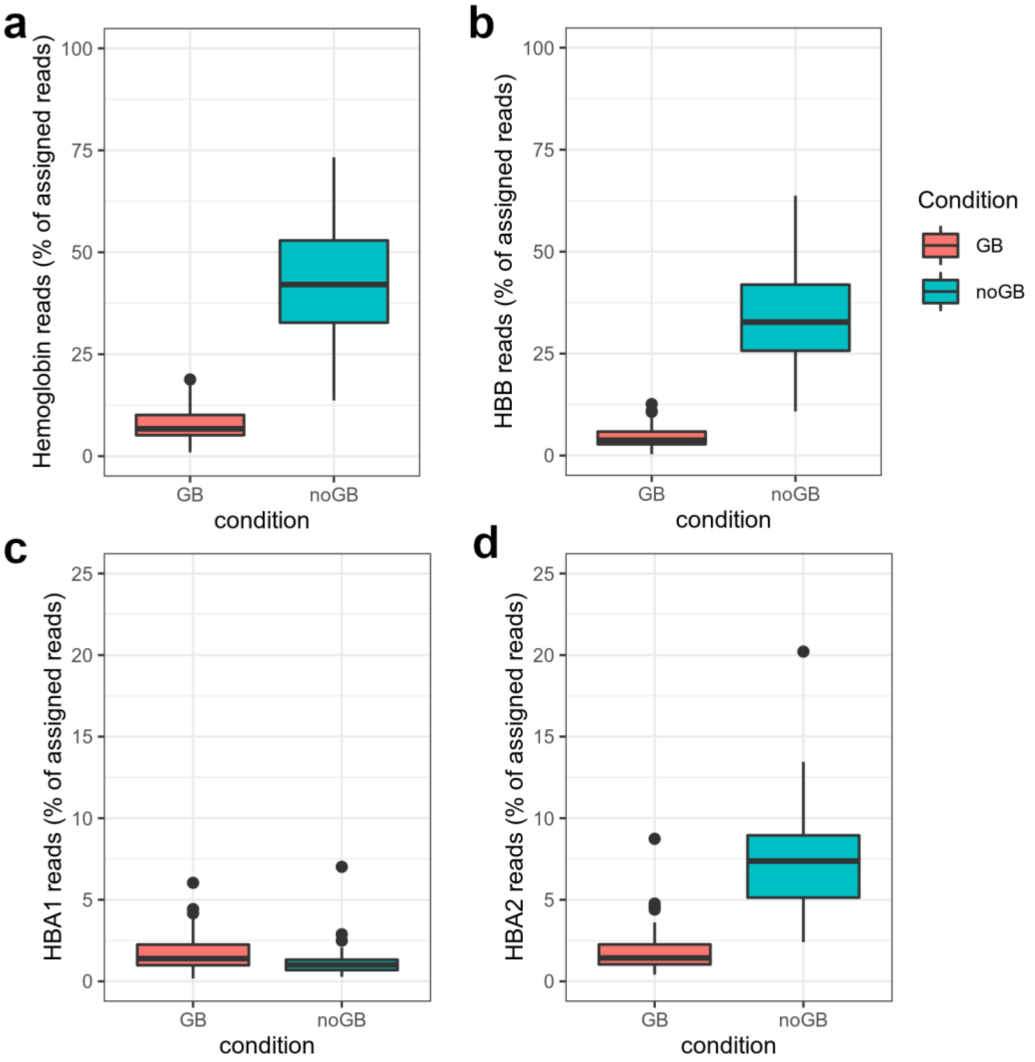

Supplementary Figure S2. HBB and HBA2 are effectively depleted. Whole hemoglobin, HBA2 and HBB are reduced in almost every samples, there is only a very limited number of outliers, for which Globin Block showed limited effectiveness. HBA1 seems not to be targeted by Globin Block, as its expression appears to increase with GB, as it is expected for non-globin transcripts.

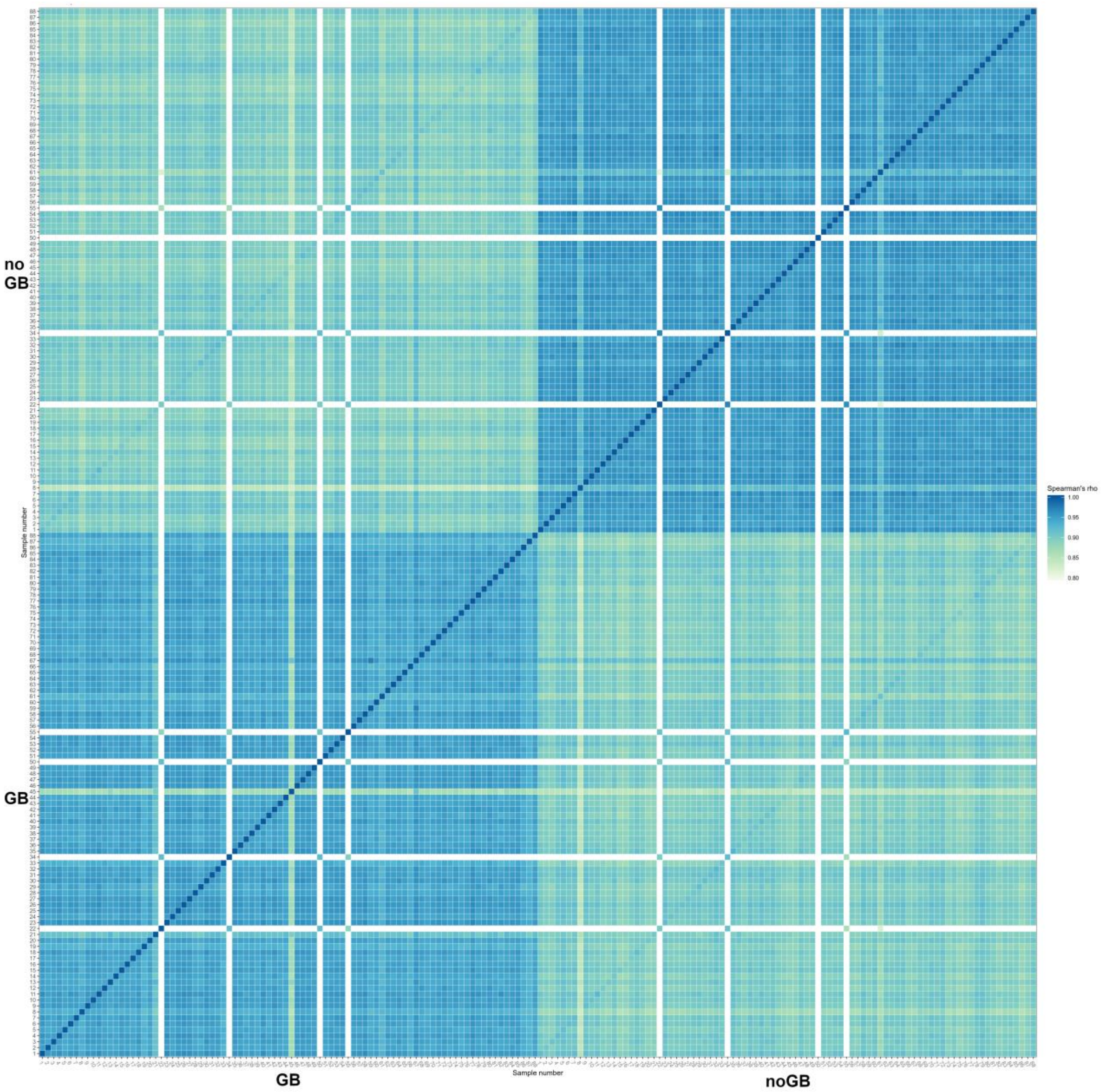

Supplementary Figure S3. Matrix of Spearman correlation coefficients of all samples of dataset 1 shows that biological identity of the samples is not the only factor. GB samples among each other and noGB samples among each other have a higher correlation than random GB and noGB samples. Although the diagonal of correct sample matches in the greenish noGB/GB quadrants is visible to the human eye, the correlation of two biologically identical samples is often not the highest in its respective row or column. Samples with very low correlations to other samples appear as white lines (which we call “outliers” in the manuscript).

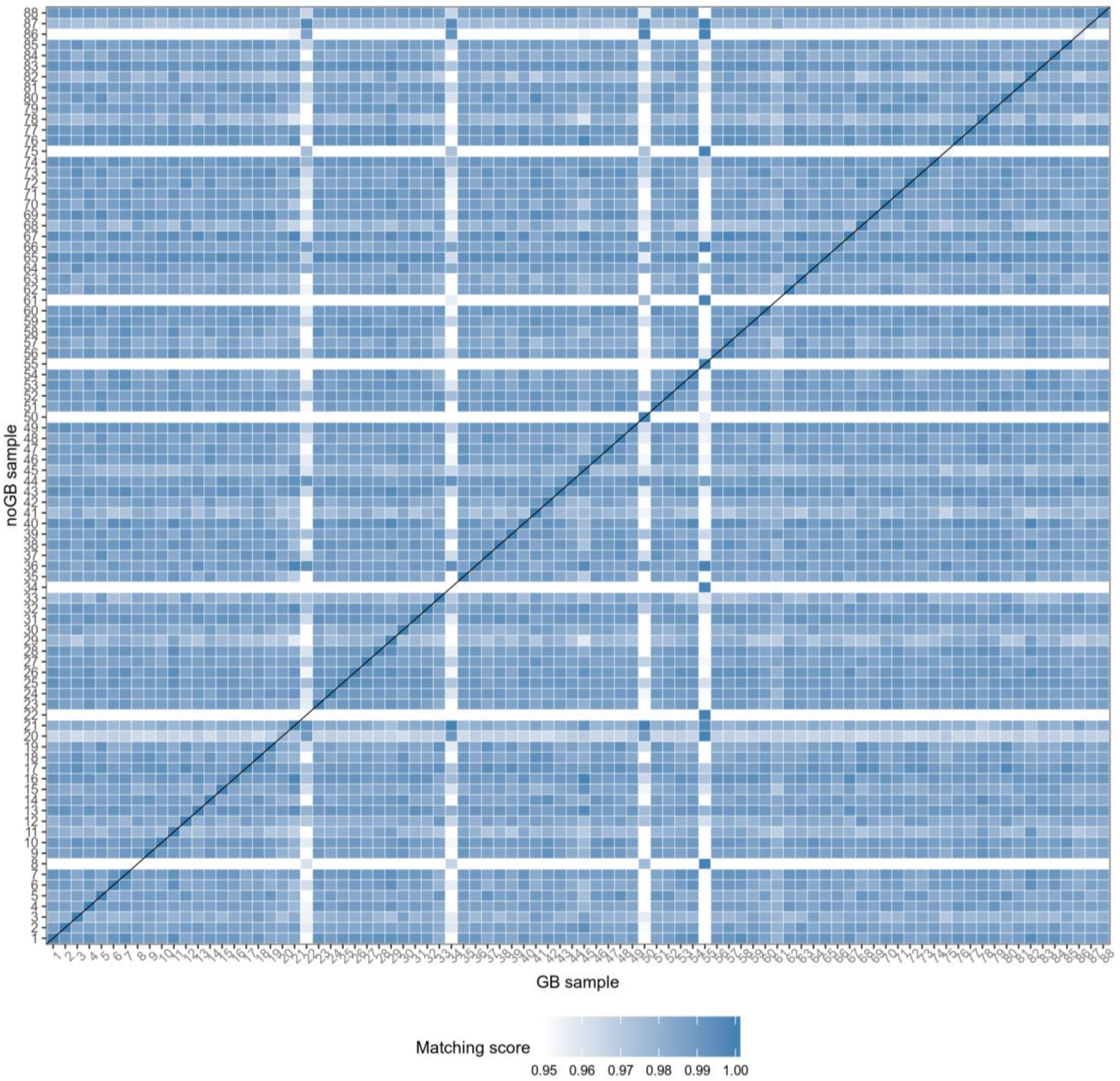

Supplementary Figure S4. Four samples, #22, #34, #50 and #55 disturb correct matching of many samples. If only one is removed, the others inherit the mismatches.

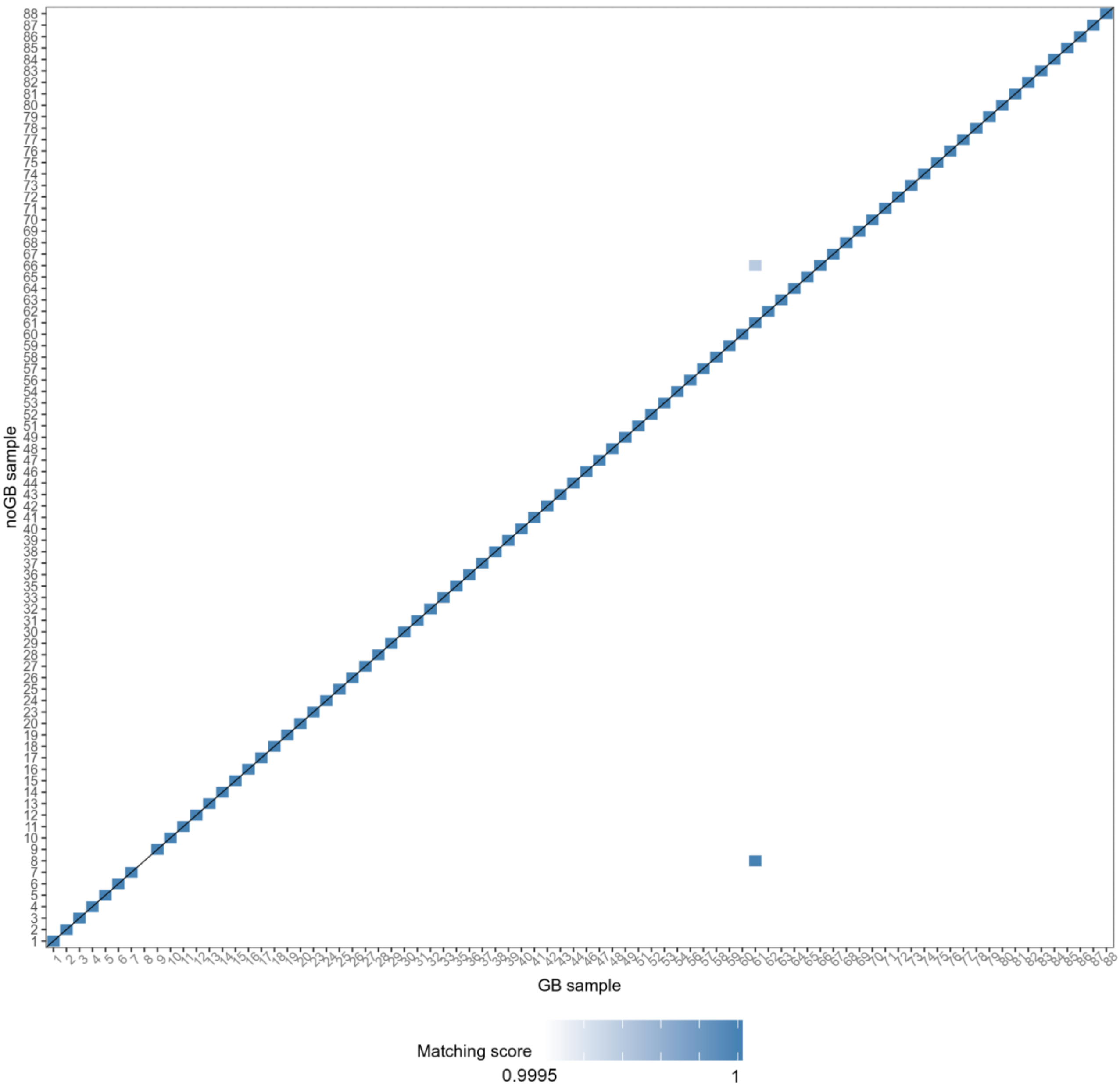

Supplementary Figure S5. After removing samples #21 and #45, matching works almost perfectly. This shows that not the scaled Spearman coefficient by itself causes the outliers.

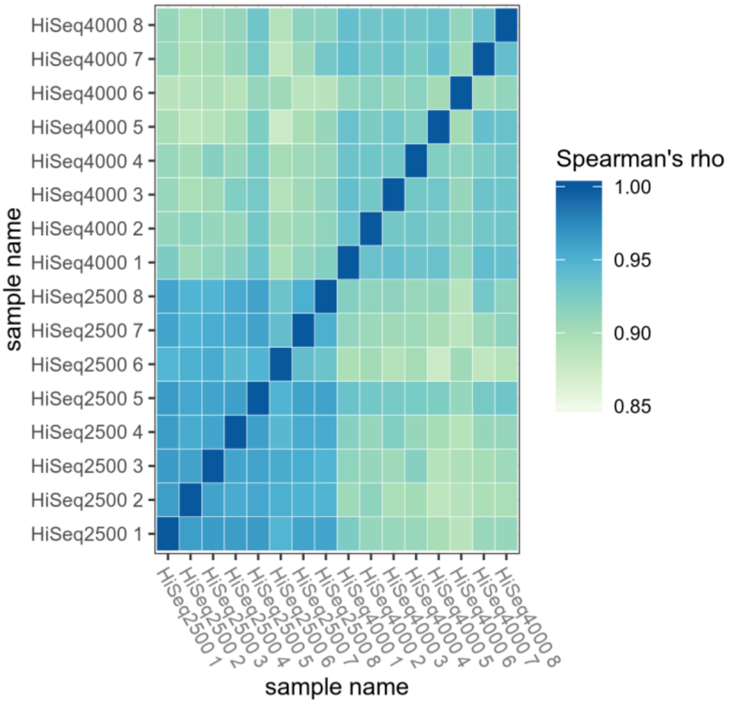

Supplementary Figure S6. Correlation coefficients of GB samples in dataset 2 are high, but do not reveal the correct sample matchings to the human eye like in Supplementary Figure S3. This indicates that reproducibility over sequencing platforms might not be given.

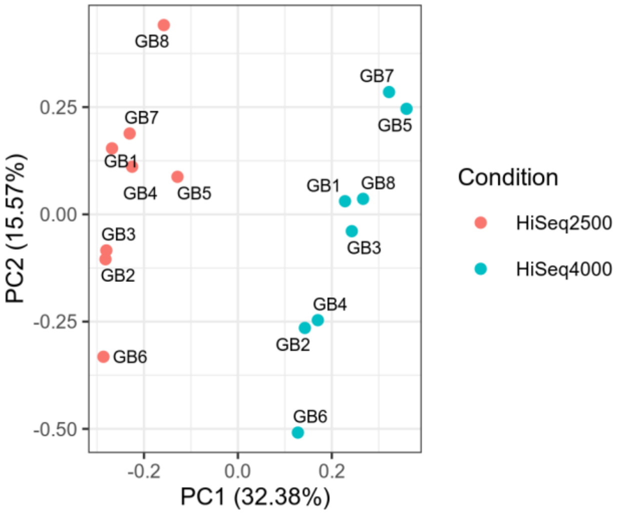

Supplementary Figure S7. PCA of HiSeq2500 and HiSeq4000 GB samples. Data points are separated by the first component.

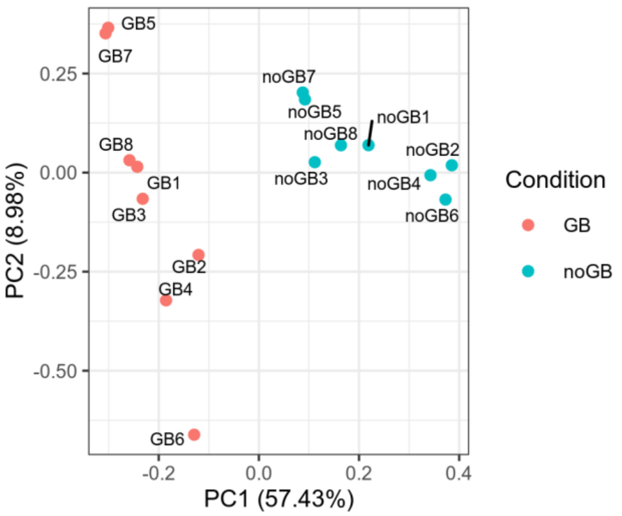

Supplementary Figure S8. PCA of GB and noGB samples on a single lane of a HiSeq4000. GB and no GB samples are separated by the first principal component. Furthermore, the very similar relative positions of the single data points in the two clusters are in line with the perfect matching of the samples in Figure 5b.

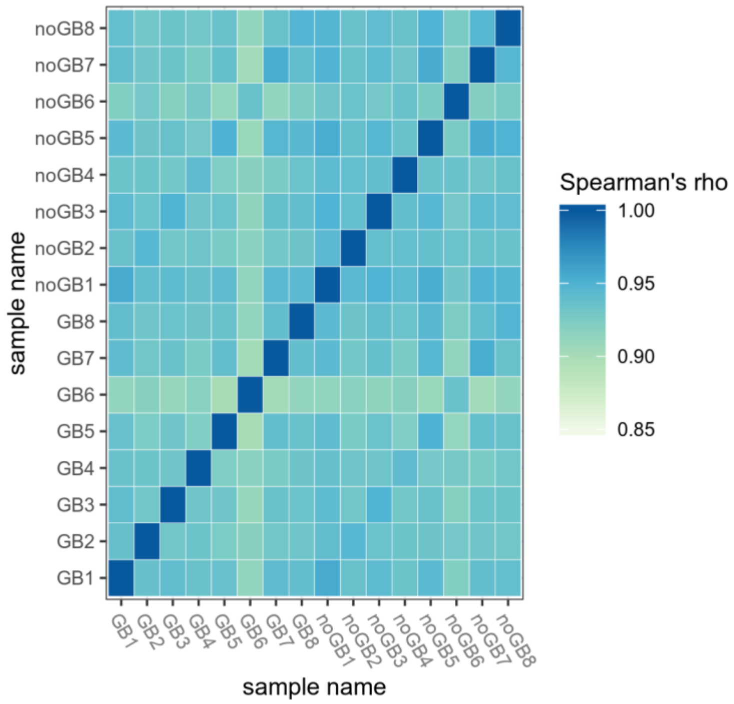

Supplementary Figure S9. Spearman correlations between GB and noGB samples from a singe lane of a HiSeq4000. In comparison to the correlation coefficients of dataset 1 (GB vs. noGB on two different lanes of a HiSeq2500, see Fig. 3, Supplementary Fig. S3), the GB vs. GB, noGB vs. noGB and GB vs. noGB correlation coefficients are similar. The correct matching diagonal (GB1/noGB1, GB2/noGB2…) is easily identifiable.
